# Assessing the potential for assisted gene flow using past introduction of Norway spruce in southern Sweden: Local adaptation and genetic basis of quantitative traits in trees

**DOI:** 10.1111/eva.12855

**Published:** 2019-08-22

**Authors:** Pascal Milesi, Mats Berlin, Jun Chen, Marion Orsucci, Lili Li, Gunnar Jansson, Bo Karlsson, Martin Lascoux

**Affiliations:** ^1^ Department of Ecology and Genetics, Evolutionary Biology Centre Uppsala University Uppsala Sweden; ^2^ The Forestry Research Institute of Sweden (Skogforsk) Uppsala Sweden; ^3^ The Forestry Research Institute of Sweden (Skogforsk) Ekebo Sweden

**Keywords:** assisted gene flow, breeding strategy, climate change, exome capture, *Picea abies*, quantitative traits architecture

## Abstract

Norway spruce (*Picea abies*) is a dominant conifer species of major economic importance in northern Europe. Extensive breeding programs were established to improve phenotypic traits of economic interest. In southern Sweden, seeds used to create progeny tests were collected on about 3,000 trees of outstanding phenotype (‘plus’ trees) across the region. In a companion paper, we showed that some were of local origin but many were recent introductions from the rest of the natural range. The mixed origin of the trees together with partial sequencing of the exome of >1,500 of these trees and phenotypic data retrieved from the Swedish breeding program offered a unique opportunity to dissect the genetic basis of local adaptation of three quantitative traits (*height*, *diameter* and *bud‐burst*) and assess the potential of assisted gene flow. Through a combination of multivariate analyses and genome‐wide association studies, we showed that there was a very strong effect of geographical origin on growth (*height* and *diameter*) and phenology (*bud‐burst*) with trees from southern origins outperforming local provenances. Association studies revealed that growth traits were highly polygenic and *bud‐burst* somewhat less. Hence, our results suggest that assisted gene flow and genomic selection approaches could help to alleviate the effect of climate change on *P. abies* breeding programs in Sweden.

## 1 INTRODUCTION

Local adaptation is pervasive and phenotypes often match their environments as demonstrated by the clinal distribution of many phenotypic traits along environmental gradients (Savolainen, Lascoux, & Merilä, 2013). Since most adaptive traits are quantitative, understanding the genetic control of quantitative traits is therefore of paramount importance to predict their evolution under environmental changes. Unravelling the genetic basis of most phenotypic traits is, however, challenging because of their complex determinism. Most phenotypic traits are indeed controlled by a large number of genetic and nongenetic factors (e.g. epigenetic factors, environmental effects), and the development of next‐generation sequencing has just begun to shed light on the complexity of their genetic architecture (e.g. Boyle, Li, & Pritchard, 2017; Wray, Wijmenga, Sullivan, Yang, & Visscher, 2018; Zeng et al., 2018). Another major finding of the last decades is the fact that local adaptation at quantitative traits is primarily caused by correlated changes in allelic frequencies at a large number of loci rather than through strong shift in allele frequencies at a few quantitative trait loci (QTL; Le Corre & Kremer, 2003; Berg & Coop, 2014). These general properties of quantitative traits imply that identifying genetic polymorphism associated with phenotypic traits or their local adaptation will be a daunting task. Consequently, we should try to optimize the use of available data, especially when working with long‐lived organisms such as trees. In the present study, we used data from the Norway spruce breeding program to study the genetic architecture of three phenotypic traits of adaptive and economic interest and assess the potential of assisted gene flow.

Climate change is rapidly altering the environment of plants and animals, especially at high latitudes (Root et al., 2003; Walther et al., 2002). In order to alleviate the impact of climate change, Aitken and Whitlock (2013) proposed to use assisted gene flow. The basic idea is that species currently adapted to dry and warm environments will be pre‐adapted to the new environmental conditions prevailing in regions that experienced colder and wetter climates until today. Hence, facilitating introgression of alleles from southern populations into more northern ones could accelerate the process of local adaptation of quantitative traits to the new climatic conditions.

Transferring material from southern latitudes to more northern ones is not a new idea, and extensive seeds transfer already took place in the past. Indeed, since the 1950s, Sweden, Norway, and to a lesser extent Finland, started to import seeds of Norway spruce (*Picea abies*) for forest reproduction material from Belarus, the Czech Republic, Romania, Germany, Slovakia and the Baltic States (Myking, Rusanen, Steffenrem, Kjær, & Jansson, 2016; Jansen, Konrad, & Geburek, 2017). As a matter of fact, we recently showed using genomic data that a very large part of the plus trees (trees of outstanding phenotype) used to establish the Norway spruce breeding program in southern Sweden in the fifties corresponded to recent introductions (Chen et al., 2019). Because of the continuous introduction of material from the rest of the natural range of Norway spruce, the Norway spruce breeding program today includes individuals from the seven *P. abies* genetic clusters (Chen et al., 2019): Alpine, Fennoscandian and Carpathian, but also central Europe (resulting from hybridization between Alpine and Carpathian clusters), central and southern Sweden (hybridization between Alpine and Fennoscandian clusters), northern Poland (hybridization between Fennoscandian and Carpathian clusters) and Russia‐Baltics (hybridization between Fennoscandian and Carpathian clusters with a strong introgression of *Picea obovata*). The aim of these introductions was twofold: (a) to obtain a large amount of seeds and (b) to take advantage of the fact that trees from lower latitudes have a longer growth period and thereby a higher yearly growth rate than local provenances when moved northwards (Clapham et al., 1998; Dormling, Gustafsson, & Wettstein, 1968; Ekberg, Eriksson, & Dormling, 1979).

The provenance and progeny tests installed in relation with the breeding program represent a great opportunity to study the genetic basis of quantitative traits and assess the amount of local adaptation. In particular, in contrast to individuals sampled in natural populations, the genotype–phenotype relationships are easier to establish as trees from different geographical areas were planted in the same environment. However, the important seed transfers that occurred in the past also imply that the Norway spruce population in southern Sweden is today highly structured and admixed, a factor that will need to be accounted for when investigating genotype–phenotype associations or when establishing training sets for genomic selection (Heslot, Jannink, & Sorrells, 2015; Grattapaglia et al., 2018).

**Figure 1:**
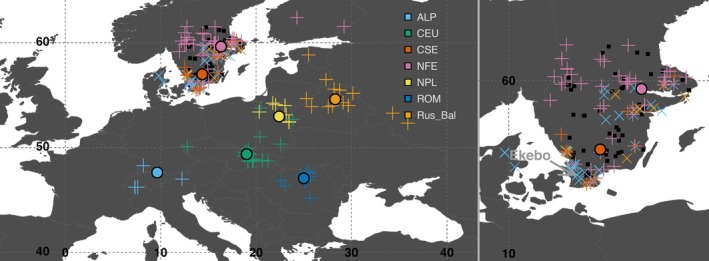
Trees original locations. Black squares are Norway spruce progeny test belonging to the Swedish breeding program. ‘Plus’ and ‘multiply’ signs are tree sampling locations, and the latter indicating a wrong assignation in available records. Discs are the centroid of the geographical coordinates of trees belonging to a same genetic cluster and having a known origin. The grey arrow (right panel) indicates the location of the trial ‘Ekebo’, where *bud‐burst* was characterized. Colours correspond to genetic clusters (Carpathian, dark blue, ROM; Alpine, light blue, ALP; Central Europe, green, CEU; Northern Poland, yellow, NPL; Russia‐Baltic, orange, Rus‐Bal; central and southern Sweden, red, CSE; Fennoscandian, pink, NFE)

In the present study, we will take advantage of these extensive past transfers and their inclusion in the breeding program to (a) assess the level of local adaptation of trees currently growing in southern Sweden and (b) investigate quantitative trait genetic architecture. A large part of the exomes of more than 1,500 trees that were used to create the Norway spruce breeding program for southern Sweden were sequenced (Figure 1). Offspring of these trees were used in progeny tests across southern Sweden in order to estimate the breeding values of their parents for three phenotypic traits of economic interest, *diameter*, *height* and *bud‐burst*. While *diameter* and *height* are related to wood production, *bud‐burst* reflects growth rhythm.

First, we reasoned that high pairwise correlations between genotype, phenotype and climatic variables at origin would not be obtained if the current genetic structure among populations were only the result of past demographic events and isolation by distance. Indeed, we detected a strong level of past local adaptation with high congruence between the clustering of genotypic, phenotypic and climatic data. Levels of productivity of southern provenances under current climatic conditions in southern Sweden are thus higher because of a longer growth period. Through genome‐wide association studies (GWASs), we then investigated both the genetic basis of Norway spruce local adaptation to climate and the genetic control of three phenotypic traits of economic interest. We identified a large number of genes involved in the response to environmental variation or in the control of quantitative traits. Especially, we showed that the genetic control of growth traits is much more polygenic than that of *bud‐burst*. Our data also highlight how traits with different patterns of geographical variation can be used to assess the impact of correction for population genetic structure in GWAS. More importantly, we argue that while data from breeding programs might sometimes be incomplete or suboptimal, they are readily available and contain a lot of valuable information for evolutionary biologists.

## 2 MATERIAL AND METHODS

### 2.1 Trees sampling

The original sampling included 1,672 samples from three related spruce species, Norway (*P. abies*), Siberian (*P. obovata*) and Serbian (*Picea omorika*) spruces (Chen et al., 2019). In the present study, only 1,545 trees from *P. abies* populations were considered (Figure 1 and Table S1). These samples came from two sampling schemes:

1. A total of 1,475 individuals were ‘plus trees’, *that is* trees selected on the basis of their outstanding phenotype to create the base population of the Norway spruce Swedish breeding program. Needles were collected on trees from Skogforsk (The Forestry Research Institute of Sweden) clonal archives. Their progeny were represented in several progeny tests across central and southern Sweden (Figure 1, black squares). The trials were established between 1978 and 1998. They all are open‐pollinated progeny trials (for estimating breeding values of the parent trees and performing backward selection). All trials were designed as randomized incomplete blocks. The number of families/parents tested in each trial varies between 30 and 1,395 and the number of replicates between 11 and 40. Finally, all trials were designed as single‐tree plots (single trial information's are reported in Appendix S1). Among those 1,475 ‘plus trees’, 560 had no clear records of their geographical origin (i.e. information on the origin of trees was missing in the archives of the breeding program, Table S1).
2. A total of 70 individuals were sampled from *P. abies* natural populations covering the main genetic domains of its distribution range (Lagercrantz & Ryman, 1990; Tollefsrud et al., 2009; Tsuda et al., 2016; Chen et al., 2019). They were used as reference when defining the origin of the 560 ‘plus trees’ whose origin was missing (Table S1).

### 2.2 Population structure analyses

The SNP data set defined by Chen et al. (2019) was used to assess population structure and to define genetic clusters of the 1,545 *P. abies* individuals.

#### 2.2.1 SNP identification

The complete procedure for SNP identification is detailed in Chen et al. (2019). Briefly, after genomic DNA extraction, 40,018 probes of 20bp long were designed to cover exons of 26,219 *P. abies* contigs (Vidalis et al., 2018). Paired‐end short reads were aligned to the reference genome of *P. abies* (Nystedt et al., 2013) using BWA‐mem (Li & Durbin, 2009). PCR duplicates were removed using PICARD v1.141 (http://broadinstitute.github.io/picard), and INDEL realignment was performed using GATK (McKenna et al., 2010) IndelRealigner. SNP calling was carried out using GATK HaplotypeCaller across all samples. After variant recalibration and filtering (≤2 reads for both, reference and alternative alleles and ≤50% coverage across individuals), 1,004,742 SNPs were retained. The original study included few individuals belonging to *P. obovata* and *P. omorika* species. Only polymorphic sites within *P. abies* were considered in the present study reducing the size of the data set to 917,107 SNPs.

#### 2.2.2 Genetic clusters definitions, inference of individuals’ unknown origin and relatedness

EIGENSOFT v6.1.4 (Galinsky et al., 2016) was used to perform principal component analysis (PCA) on the genetic variation of *P. abies* and to define subsequent genetic clusters based on 399,801 unlinked noncoding SNPs (pairwise LD ≤ .2 and FDR value ≥ .05, after haplotype phasing using MACH v 1.0, Li, Willer, Ding, Scheet, & Abecasis, 2010). Geographic origin of the 560 individuals for which no confident records of geographical origin were available was then assessed based on their genotype similarity to ascertained individuals. *P. abies* individuals of known origin were first grouped into seven major clusters based on genetic clustering results and their origin. These individuals were then used as a training set in a ‘Random Forest’ regression model (‘randomForest’ v4.6‐14 package, Liaw & Wiener, 2002, R software v.3.3.1, R Core Team, 2019). The first five components of the PCA analysis were used for model fitting to classify the unknown individuals into each of the seven clusters. Fivefold cross‐validation was performed for error estimation. Briefly, the ‘training’ data set made of individuals with known origin is randomly divided into five groups or folds. Then, one group is extracted from this training data set and serve as a validation set, and the model is fitted to the remaining groups and validated on the validation set. Individuals of ‘unknown’ origin were then assigned to the various genetic clusters defined from individuals from known origin. The whole regression process was repeated 1,000 times in order to estimate the confidence of each assignment.

Finally, for GWAS, the individual relatedness (kinship) matrix was estimated using the ‘Centered IBS’ method (Endelman & Jannink, 2012) implemented in the TASSEL software (v.5.2.38, Bradbury et al., 2007).

### 2.3 Phenotypes

Breeding values (BVs) for two growth traits *diameter* and *height* and measures of *bud‐burst* were extracted from the records of Skogforsk for 763, 808 and 834 ‘plus trees’, respectively. Complete records of the three traits were available for 712 of those trees but the origin was known for only 279 of them. Briefly, for *height* and *diameter* more than 15 progenies of each ‘plus tree’ were planted in up to five progeny tests per tree scattered across central and southern Sweden. For *height* and *diameter*, the BVs were then computed, for each progeny test, using mixed linear model and BLUPs (best linear unbiased predictors) methodology through a restricted maximum likelihood approach from the following statistical model:

(1) $$y_{\mathrm{ijk}}=\mu +b_{i}+f_{j}+\varepsilon_{\mathrm{ijk}}$$

where for a given trait, *y_ijk_* is the observation for individual *k* from family *j* in block *i*, *μ* is the mean of the trait, *b* is the fixed effect of block, *f* is the random effect of family with a normal distribution $N\left(0,\sigma_{\varphi }^{2}\right)$ and *ε* is the error term with a normal distribution $N\left(0,\sigma_{\varepsilon }^{2}\right)$. BVs were then reported as relative percentages to the mean. Therefore, a value of 100 corresponds to the average BV and a relative BV of 110 thus indicates that the given genotype has a BV 10% higher than the average. For a given genotype, the average BV across the different progeny tests was then considered. *bud‐burst* was measured using Krutzsch scale (Krutzsch, 1973), ranging from 0 (no burst) to 9 (full development of the needles) from a single clonal archive and transformed to normal scores based on midpoint values of the cumulative frequency distribution (Danell, 1991) before analysis.

#### 2.3.1 Inference of missing phenotype

Missing phenotypes (*N* = 782, *N* = 737 and *N* = 711, respectively, for *diameter*, *height* and *bud‐burst*) were inferred from genotypic data using the ‘genomic selection’ method implemented in TASSEL software (v.5.2.38 Bradbury et al., 2007; Zhang et al., 2010). Briefly, each trait was considered independently and the missing breeding values for a given trait were estimated using a mixed model that included a population structure matrix as fixed effects and a kinship matrix as random effects to capture the covariance between genotypes. In other words, the BLUPs of individuals whose phenotype is missing are imputed from the phenotypes of closely related individuals. Fivefold cross‐validation was performed for accuracy estimation (20 iterations each).

### 2.4 Relationships between phenotype and ancestral environment

The relationship between tree origins and their phenotypes was estimated using the following generalized linear model (GLM):

(2) $$y_{\mathrm{ik}}=\mu +o_{i}+\varepsilon_{\mathrm{ik}}$$

where for a given trait, *y* is the observation for genotype *k* (BVs for *height* and *diameter* or normal score for *bud‐burst*) from origin *i*, *μ* is the mean value of the trait, and *o* is the tree‐origin specific fixed effect (a factor with seven levels corresponding to the seven genetic clusters), and *ε_ik_* is the error term, $N\left(0,\sigma_{\varepsilon }^{2}\right)$. The significance of the difference between factor levels (genetic clusters) was computed from the complete model (2) using likelihood ratio test (LRT). Factor levels were grouped if no significant difference (*p* > .05) was identified, and tree origin effect of reduced levels was further assessed as a simplified model (2).

#### 2.4.1 Ancestral environment characterization

Data for 19 bioclimatic variables (monthly averages for the 1970–2000 period, 10 arc minute resolution, ~340 km^2^) were downloaded from the online WorldClim database (v.2.0 http://worldclim.org/, Fick & Hijmans, 2017). Two additional variables were computed from these data, the summer heat‐moisture index (SHM) and annual heat‐moisture index (AHM). A measure of yearly photoperiodic amplitude (ΔDL) was also computed as the difference between the average day length in June and the average day length in January (see Table S3 for the complete list and details). For trees of unknown origin, bioclimatic data were extracted at the location corresponding to the centroid of the geographical coordinates of trees belonging to the same genetic cluster and having a known geographical origin (Figure 1, large ‘plus’ signs).

### 2.5 Outlier detection and genotype–phenotype–environment associations

#### 2.5.1 Genotype ‐ environment association

The Bayenv software (v.2, Coop, Witonsky, Rienzo, & Pritchard, 2010; Günther & Coop, 2013) was used to estimate correlations between allele frequencies at individual loci and bioclimatic variables, while accounting for population structure. A Bayesian mixed linear model, considering bioclimatic variables as fixed effects and a variance–covariance matrix of allele frequencies as random effect (to capture shared polymorphism due to populations common history), was fitted to population allele frequencies. In parallel, Spearman's rank correlation coefficient, rho, was also computed from standardized allele frequencies, from which the covariance structure among populations was removed.

Forty‐eight *P. abies* populations were defined by grouping trees from close geographic origins and belonging to the same genetic cluster (≥5 trees per population, several population per genetic cluster, circles). Note that only trees with a known origin were considered as the populations were defined from geographic coordinates (777 trees). Twenty variance–covariance matrices were estimated from 20,000 noncoding, and unlinked SNPs randomly sampled from the 399,801 noncoding and unlinked SNP data set. The average matrix across the 20 runs was then considered in the model. Finally, for each population, bioclimatic data for each tree location were averaged. For each climatic variable, the following filtering (based on Bayes factor and Spearman's rho) was applied to retain only the most relevant SNPs: (a) the SNPs were ranked according to their Bayes factor (BF) and a SNP was retained if its BF > 150 (very strong strength of evidence according to Kass and Raftery (1995) or, if its BF > 20 (strong strength of evidence) and was within the .1% highest BF; (b) in parallel SNPs were ranked according to Spearman's rho and only those that were satisfying the first criteria and were within the 1% highest absolute rho were conserved for further analysis, as recommended by Günther and Coop (2013).

#### 2.5.2 Genotype–phenotype association

For each trait independently, the additive allelic effect of each SNP on the phenotype and the corresponding standard error were estimated through a linear mixed model considering population structure (first three principal components of a SNP‐based PCA) and individual relatedness (kinship matrix). The analysis was performed through a compressed mixed linear model (Zhang et al., 2010) implemented in the R package GAPIT (Lipka et al., 2012). For this analysis, only bi‐allelic SNPs with a minimum allele frequency > .05 and a minimum number of individuals per genotype of 10 were considered.

The statistical significance of the SNP associated to the three phenotypic traits was investigated using a recently developed Empirical Bayes approach for adaptive shrinkage (Stephens, 2017) implemented in the R package *ashr* (Stephens et al., 2018). Traditional false discovery rate (FDR, Storey, 2003) methods are based on the sole *p‐*values. In contrast, *ashr* uses both allelic effect sizes and their standard errors. It models the GWAS results as a mixture of SNPs that have a true effect size of exactly zero and SNPs that have a true effect size that differs from zero. The ‘local false sign rate’, *lfsr*, which refers to the probability of getting the sign of an effect wrong, is then used as a measure of significance and to compute *s*‐values (Stephens, 2017), which are the analogues of Storey's *q*‐values (Storey, 2011). The ‘local false sign rate’ is therefore more robust to errors in model fit than FDR (Stephens, 2017).

#### 2.5.3 Gene function and enrichment test

Gene ontology (GO) enrichment was performed using the ‘top GO’ R package (v2.26.0; Alexa & Rahnenfuhrer, 2010). Annotation from ConGenIE (the Conifer Genome Integrative Explorer, http://congenie.org/) was used as reference (i.e. custom input), and all the GO terms were conserved (nodeSize parameter = 1). For the various lists of candidate genes defined through both SNP‐environment and SNP‐phenotype analyses, enrichment of genes in particular GO terms biological processes (BP) was assessed using ‘weight’ algorithm and Fisher's exact test (*p* < .05). Finally, the REViGO software (Supek, Bošnjak, Škunca, & Šmuc, 2011) was used to remove GO terms redundancy and to cluster the remaining terms in a two‐dimensional space derived by applying multidimensional scaling to a matrix of the GO terms semantic similarities (default parameter setting: allowed similarity = .7, SimRel to measure the semantic similarity, UniProt as database). The Cytoscape software v3.6.1 (Shannon et al., 2003) was then used to visualize GO terms networks.

## 3 RESULTS

### 3.1 Ancestral environment is a strong predictor of phenotype in Norway spruce

**Figure 2:**
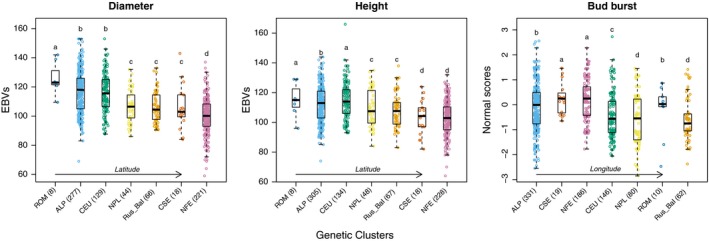
Influence of trees origin on phenotype. *Diameter*, *height* (breeding values, BVs) and *bud‐burst* (normal scores) values are represented for the different genetic clusters (Carpathian, dark blue, ROM; Alpine, light blue, ALP; Central Europe, green, CEU; Northern Poland, yellow, NPL; Russia‐Baltic, orange, Rus‐Bal; central and southern Sweden, red, CSE; Fennoscandian, pink, NFE). The genetic clusters are ordered regarding latitude for *height* and *diameter* and given longitude for *bud‐burst*. The number of trees belonging to each genetic cluster is given within parentheses. Letters represent the levels of significance

Trait values estimated from progeny tests across southern Sweden were used to assess the influence of tree origin (genetic cluster) on phenotypes. All three traits, *diameter*, *height* and *bud‐burst,* differ among genetic clusters (Model 2, *f* = 39, *df* = 6, *p* < .001; *f* = 20, *df* = 6, *p* < .001; *f* = 12, *df* = 6, *p* < .001, respectively, for *diameter*, *height* and *bud‐burst;* Figure 2). Trees from the Alpine and Carpathian domains or from Central Europe tended to be bigger than trees from more northern domains. For instance, trees originating from Romania were, on average, ~25% larger and ~13% taller than trees from Fennoscandia. Patterns of variation in *bud‐burst* differed markedly from patterns of variation in *height* or *diameter* as *bud‐burst* mainly decreased along longitude, *that is* along a continentality gradient in Europe (Figure 2).

Climatic variables at tree original locations were then used to characterize the environment at origin and investigate environment–phenotype relationships. Unfortunately, due to missing records, tree origin and phenotype information were both available for only 279 trees. Both data sets were thus completed by considering bioclimatic data at the location corresponding to the centroid of the geographical coordinates of trees belonging to the same genetic cluster and having a known origin (Figure 1, large ‘plus’ sign) and by imputing missing phenotypes using linear mixed models (see Section 2 ‘Missing phenotype inference’). Please note that both geographic origin and at least one phenotypic trait were inferred for only 198 trees (~13%), and for the vast majority of the trees, either the origin or the phenotypic traits measures were available. Correlations between observed and predicted phenotypes were strong (Spearman's *ρ* = .99 for *diameter* and *height* and *ρ* = .83 for *bud‐burst*, Figure S1), but accuracy was much higher for *diameter* and *height* (.52 and .41, respectively) than for *bud‐burst* (.27). This provided us with a complete data set for the 1,543 trees.

**Figure 3:**
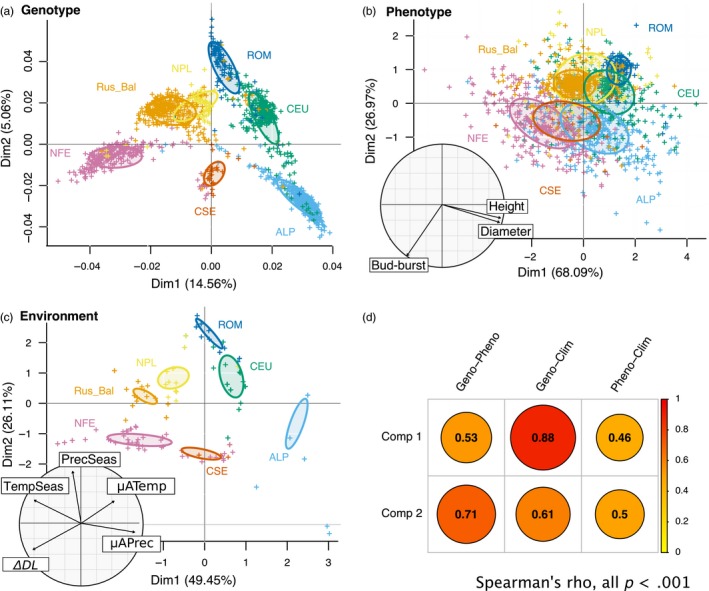
Pattern of variation of trees genotype, phenotype and original environment. Principal component analyses (PCA) based on (a), SNPs data (modified from Chen et al., 2019), (b) Phenotypic data and (c), climatic variables of the populations of origin (see Table S3 for climatic variable details). Correlation circles for phenotypic (b) or climatic variables (c) used for the respective PCA analysis are also represented. Colours correspond to genetic clusters (Carpathian, dark blue, ROM; Alpine, light blue, ALP; Central Europe, green, CEU; Northern Poland, yellow, NPL; Russia‐Baltic, orange, Rus‐Bal; central and southern Sweden, red, CSE; Fennoscandian, pink, NFE). Panel (d) represents Spearman's correlation coefficient, *r*, between principal component 1 (Comp 1) or 2 (Comp 2) of the various PCA, disc diameters are proportional to the corresponding correlation coefficient

Genotypic (~400 K SNPs), phenotypic (*diameter*, *height* and *bud‐burst*) and environmental variables were each then described through PCA (‘ade4’ R package *v.1.7‐10*, Chessel, Dufour, & Thioulouse, 2004). The latter were characterized by annual mean and seasonality of precipitation (μAPrec and PrecSeas, resp.) and of temperature (μATemp and PrecTemp, resp.), as well as by an indicator of photoperiod (ΔDL; see Table S3 for more details). Strikingly, genotype, phenotype and environment data presented very similar clustering patterns (Figure 3a–c) and the principal component coefficients of the different PCA were highly correlated (Figure 3d). To control whether such strong correlations were not due to the fact that many individuals shared the same climatic data, we reanalysed the data by randomly sampling one individual at each location, thereby avoiding any redundancy in bioclimatic information. Spearman's correlation coefficients were as strong as with the complete data set though the associated *p*‐value increased due to a much smaller data set (*N* = 103), but remained highly significant (Table S2). We also checked the influence of the inferred data on this pattern by considering only trees with complete records (*N* = 279) or trees of known origin with at least one phenotypic trait inferred (*N* = 685). In both cases, we retrieved the same pattern as with the complete data set (Figure S2a,b), but, as expected, the intensity of some correlations for the PCA with only 279 trees was lower even though the correlations remained significant.

Further, a more thorough investigation of the phenotype–environment relationships (21 climatic variables considered, see complete list in Table S3) showed that *diameter* and *height* decreased mainly along a South to North latitudinal gradient (Pearson's *r* = −.62 and −.47, respectively, all *p* < .001) and were thus strongly associated to climatic variables following this gradient (e.g. ΔDL: *r* = −.61 and −.46; summer heat‐moisture index, SHM: *r* = −.54 and −.38; annual precipitation, μAPrec: *r* = .50 and .31; all *p* < .001 Table S3 and Figure S4 for the complete analysis). On the other hand, *bud‐burst* followed both a latitudinal gradient (South to North, *r* = .39, *p* < .001) and a longitudinal gradient (West to East, *r* = −.47, *p* < .001). It was thus more associated with climatic variables reflecting continentality (e.g. PrecSeas: *r* = −.54; mean diurnal temperature range, μRangeDuir: *r* = −.52; annual temperature range, ARangeTemp: *r* = −.39; all *p* < .001. See Table S3 and Figure S3 for the complete analysis).

### 3.2 Genotype–environment association analysis revealed close link to environment of origin despite strong population structure

In order to identify loci underlying local adaptation in Norway spruce, different populations were defined according to tree original locations (i.e. trees from close geographic origins belonging to the same genetic cluster were grouped, and several populations per genetic cluster have been defined, Table S4, ‘circles’); note that only trees with a known origin were used in the present analysis (48 populations, 777 trees, Table S4). Climatic variables were then used as fixed factors in independent mixed linear models (MLMs) accounting for population structure (Bayenv software v.2, Coop et al., 2010; Günther & Coop, 2013), to explain SNP frequencies variation across populations (Figure S4).

After stringent filtering steps to control for false positives (see Section 2 ‘SNP‐Environment relationships’), many SNPs were found to be significantly associated with environmental variables (min = 46 SNPs for Temp_1, annual mean temperature and max = 344 SNPs for Prec_4, total precipitation of the driest month, Table S5). Approximately 25% of these SNPs belong to intergenic regions, a half belongs to introns and the remaining quarter belongs to exons (Table S5; SNP annotations and transcript descriptions are given in Appendix S2). For each climatic variable, the redundancy varied a lot from 0% to ~65%, meaning, for the latter, that two‐third of the candidate SNPs are related to the same transcripts (Table S5). Despite such a degree of redundancy, many genes were found to be associated with each climatic variable (min = 43 genes for Temp_1, annual mean temperature, and max = 148 genes for photoperiod, Table S5). On average, a larger number of transcripts were associated with precipitation‐related variables (*μ*_prec_ = 87, mean overlap between variables 44%, Table S6a) than with temperature‐related variables (*μ*_temp_ = 68, overlap 12%, Welch's two‐sample *t* test, *t* = −4.9, *df* = 11, *p* < .001) or moisture (*μ*_moist_ = 68, overlap 38%). The largest group consists of transcripts associated to photoperiod (*N* = 148, Table S5). Importantly, the present study demonstrates that climatic variation creates a widespread selective pressure across the Norway spruce genome as nonoverlapping categories of genes were associated to different climatic variables. For instance, the average overlap between genes involved in response to temperature variables and those involved in response to precipitation was only 5% (Table S6b).

However, this limited overlap at the gene level masks a much more important overlap at the functional level as many of these genes tend to be involved in the same BP as shown by the strong overlap between GO terms (Appendix S2). The smallest overlap was between photoperiod and moisture index, 15%, and the largest, 72%, expectedly, was for precipitation and moisture (Figure S5a).

### 3.3 Growth traits and bud‐burst have different genetic architecture

To characterize the genetic architecture of the different phenotypic traits and to identify SNPs involved in their control, GWASs were conducted with MLMs. Population structure was considered by including the three first principal components of a SNP‐based PCA as fixed effects and a kinship matrix as random effect in the MLM; note also that only trees with a measured phenotype (i.e. not inferred) were included in these analyses (763, 808 and 834 trees, respectively, for *diameter*, *height* and *bud‐burst*).

**Figure 4:**
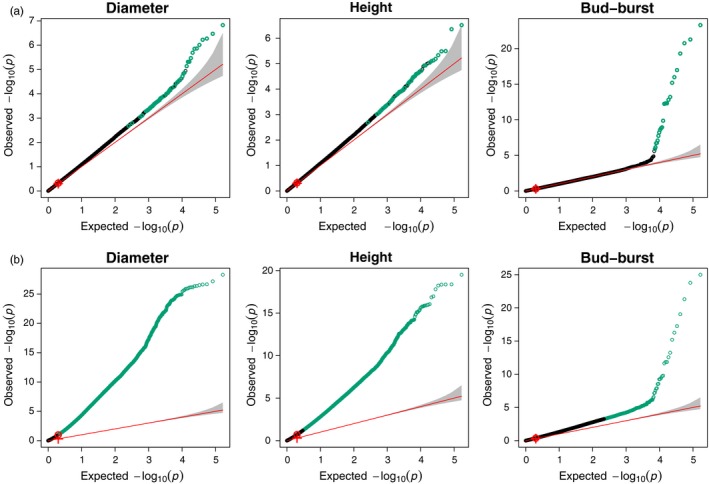
Q–Q plot of observed versus expected *p*‐values. For each phenotypic trait, −log_10_ *p*‐values, controlling for population structure (a) or not (b), are represented as a function of expected −log_10_ *p*‐values assuming a uniform distribution. The red line is the one to one quantile, line and the grey area is the 95% confidence intervals around it. The red circle and the ‘plus’ sign represent the medians of observed and expected *p*‐values, respectively. Significant corrected *p*‐values (*s*‐value < .1, see text) are coloured in green

*Diameter* and *height* had a highly polygenic control as no <180 and 175 SNPs, respectively, had a significant effect on trait values (*s*‐value < .1, *s* is the analogue of *q*‐value for *false sign rate* detection, see Section 2 ‘SNP‐Phenotype relationships’, Table S5 and Figure 4a). In striking contrast, a mere 32 SNPs were detected for *bud‐burst*. These SNPs affected more than 130 different genes for growth traits (~20% redundancy) but only 15 genes for *bud‐burst* (~50% redundancy).

Moreover, the phenotypic correlations between traits are due to pleiotropic effects at the genotypic level (Table 1 and Figure S6). For instance, tree height and diameter were strongly correlated (Spearman's *ρ* = .86) and such a correlation was due to correlation between SNP allelic effect sizes (*ρ* = .91 and .93, when considering SNPs significant for *height* or SNPs significant for *diameter*, respectively). The pattern for *bud‐burst* was different as SNPs involved in its control had a strong influence on both *height* and *diameter* (*ρ* = −.92 and *ρ* = −.91, respectively), but the converse was not true (*ρ* = −.36 and *ρ* = −.05).

**Table 1:** Linear regressions between either trait values or between allelic effect sizes

Trait A	Trait B	Phenotypic			All SNPs			A significant SNPs			B significant SNPs		
		*r* ^2^	*t*	*df*	*r* ^2^	*t*	*df*	*r* ^2^	*t*	*df*	*r* ^2^	*t*	*df*
Height	Bud‐burst	.12	−15	1,553	.01	−43	161,112	.12	−5	173	.9	−16	28
Diameter	Bud‐burst	.08	−12	1,553	0	−26	161,112	.01	−2^ns^	178	.9	−16	28
Height	Diameter	.72	63	1,553	.64	539	161,112	.95	58	173	.95	59	178

Note

*r* ^2^: Adjusted *R*‐squared; *t*: *t*‐statistic value, all are significantly different from 0, *p* < .001 unless indicated (ns); *df*: degree of freedom.

Finally, as for SNP related to environment, genes associated to the variation in phenotypic traits belong to BP, functions, pathways and network expected for the trait under consideration. For instance, genes involved in the control of phenotypic traits are associated to GO terms such as, to name a few, regulation of auxin metabolism, response to light and photoperiodism, gravitropism, cell growth or organs development (Appendix S2). Furthermore, in contrast with climatic variables, at the network scale, GO terms associated only with *diameter* and those associated only with *height* tended to belong to the same functional clusters (Figure S5b).

## 4 DISCUSSION

Norway spruce (*P. abies*) is a dominant conifer species of major economic importance in northern Europe. Extensive breeding programs were established to improve phenotypic traits of interest, focusing on productivity, wood quality and resistance to pathogens. Here, genetic and phenotypic information was collected on more than 1,500 trees of outstanding phenotype (‘plus trees’) that were used to establish the Swedish breeding population. Some of these trees were of local origin, but many corresponded to recent introductions from the rest of the natural range. In the present study, we demonstrated that these data present a unique opportunity to study the genetic basis and the role of local adaptation in the control of quantitative traits. This last point is crucial for breeders and forest managers as it provides them with a mean to assess the potential of assisted migration as a strategy to mitigate the impact of climate change on forest productivity and health.

### 4.1 Caveats and solutions

We used breeding values for two phenotypic traits, *height* and *diameter,* that were collected from different series of progeny tests of the Swedish breeding program. While it is unquestionable that breeding programs are a treasure trove for biologists, working from data originating from series of trials planted in the early 80s includes some serious challenges.

First, the data are heterogeneous. Indeed, the phenotypic data were collected on trees that were planted in progeny tests located at different latitudes in Sweden and the age of the trees at measurement varied across trials (from 6 to 15 years old for *height* and from 9 to 15 for *diameter*). A part of that variance was considered when computing the breeding values within trials as trees from the same trial were of the same age and obviously faced the same environment, but this nonetheless neglected genotype by environment interactions and did not remove the age variation across trials. In some trials, breeding values for height were computed with five‐year intervals and were highly similar (*r* ^2^ ~ .8, data not shown) and genotype by environment (GxE) interactions are known to be weak in *P. abies* breeding program in central and southern Sweden (Berlin, Jansson, & Högberg, 2015). The heterogeneity introduced by these two effects should thus be somehow limited. Finally, the trees belonged to different trial series, a trial series being a set of progeny tests comprising the same individuals. If the average BVs across a trial series is used, then GxE interactions are to some extent included. However, it should be pointed out that BVs from different trial series are not strictly speaking comparable as they were analysed separately. As the BVs from each trial series were compiled and used as one complete data set, there may be a bias. Such a bias would have been avoided if all the trial series had been evaluated simultaneously. The BVs would then have been truly comparable on the same scale, but this was unfortunately not possible at the time the present study was initiated. Interestingly, while this heterogeneity will certainly have weakened the clustering of the individuals based on their phenotypic data, it did not erase it altogether. Hence, our results are conservative.

Second, the data are incomplete: for roughly a third of the data set, the exact coordinates of the origin of some of the samples were unknown and phenotypic data were missing for about half of the trees. These difficulties were circumvented by using a large‐scale SNP data set and supervised machine‐learning algorithm to precisely assign each genotype to a given geographic origin (accuracy > .92, see Chen et al., 2019) and to infer the phenotypic values for trees for which records were lacking (genomic selection). Despite heterogeneity in the phenotypic data, the method was accurate enough (e.g. >50% for *diameter*) to provide us with a complete data set (~1,500 trees) for studying phenotype–environment relationships.

Re‐sequencing technologies are continuously developed, and their efficiency keeps increasing. They are now affordable (and prices are still decreasing), and it is now possible to obtain genomic data for a large number of individuals. Thanks to new statistical approaches, mostly based on machine learning (see Schrider & Kern, 2018, and examples within), it is now possible to overcome issues often encountered with such large and long‐term survey data sets such as incompleteness. Breeding programs thus represent a valuable and still underused source of study material for evolutionary biologists. This is especially true for forest trees, as progeny tests and common gardens require extensive space and need to be measured over long periods of time, something that cannot easily be done today within universities or research institutes; in our case, the trials were spread all over southern Sweden and some were started half a century ago.

### 4.2 Population structure, local adaptation and genetic architecture of quantitative traits

Animal and plant species are known to have undergone cycles of contractions and expansions as a result of successive glacial and interglacial periods (Bennett, 1997). The contraction phase is responsible for reproductive isolation between refugial area, which, in association with bottlenecks (reduction of the effective size of a population), can lead to a strong divergence between populations or even speciation events (Petit et al., 2003). During the expansion phase, secondary contacts between the genetic entities can occur, resulting in introgression that can play a major role in the evolution of species (see Arnold, 2004 and references within). Re‐colonization also involves facing different environments, and natural selection also played a role in the current distribution of species (Saccheri & Hanski, 2006). The phenotypes of individuals are therefore the result of a complex interplay between demographic history of populations and local adaptation. Because (re)‐colonization routes often followed environmental gradients, these two effects are generally confounded and disentangling the role of each in trait evolution remains challenging (Gaggiotti et al., 2009).

In *P. abies*, contraction phases resulted in three strongly differentiated genetic clusters, a northern domain in Fennoscandia and two southern domains in the Alps and the Carpathians, that have been amply documented (Acheré, Favre, Besnard, & Jeandroz, 2005; Borghetti, Glannini, & Menozzi, 1988; Chen et al., 2018; Heuertz et al., 2006; Lagercrantz & Ryman, 1990; Tollefsrud et al., 2009; Tsuda et al., 2016). These three genetic clusters had a major impact on phenotypic divergence as illustrated in our study and in the seminal work of Lagercrantz and Ryman (1990). In the latter, the authors analysed 48 Norway spruce provenances at both 22 allozyme loci and seven morphological characters describing seed, growth and phenology. They analysed both allozyme and phenotypic variations with principal components analysis and, as in our case, observed a striking similarity between the two resulting plots indicating that population history had a strong impact on the divergence of phenotypic traits.

In our case, the strongest correlation was found between genotype and climatic data at trees origin, which roughly reflects geography. This is expected since Norway spruce is known to present a relatively strong population structure, with the different populations located in different selective environments. Genotypic data thus capture both demographic history and local adaptation. If the correlation between genotypic data and climatic data were only reflecting past demography and isolation by distance, we would not expect to also detect a correlation between both genotypic data and phenotypic data, on the one hand, and between phenotypic data and climatic data, on the other hand. However, both were also high and significant (Figure 3d). Correlations involving phenotypic data were the lowest, probably because the phenotypic data were also the noisiest and are inherently more complex than environmental variables. As stated above, even if limited, both GxE interactions and differences in trees age at measurement between trials have probably introduced heterogeneity in the phenotypic data sets. In any case, the fact that environmental variables at the locations of origin of the different clusters show the same clustering as both phenotypic traits *and* genetic polymorphism strongly suggests that this divergence is not entirely neutral and may reflect local adaptation (Savolainen et al., 2013).

This agrees with other studies of local adaptation in forest trees that have all concluded that local adaptation is common in forest trees despite extensive gene flow (e.g. Avia, Kärkkäinen, Lagercrantz, & Savolainen, 2014; Chen et al., 2012, 2014; Lind et al., 2014; Yeaman et al., 2016). In a simple, single‐locus model of local adaptation, one would have expected low levels of local adaptation when gene flow is strong (Bulmer, 1972). This apparent paradox was first explained by Le Corre and Kremer (2003; see also Le Corre & Kremer, 2012; Kremer & Le Corre, 2012). Their model was later extended by Berg and Coop (2014) which, in brief, showed that high differentiation between populations at quantitative traits will not result from large change in allele frequencies at a limited number of loci but instead will follow from coordinated small changes in allele frequencies at a myriad of loci, each of small effect, underlying the variation in the quantitative traits.

We indeed found that the three traits used in the present study were highly polygenic, albeit *height* and *diameter* appeared more polygenic than bud‐burst. The latter is unlikely to be a consequence of differences in heritability among traits as *bud‐burst* tend to have a higher heritability than *height* and *diameter,* and therefore, all things being equal it should be easier to detect loci associated with bud‐burst than to *height* and *diameter* (Hannerz, 1998 and references therein). Incidentally, our results also have important consequences for the estimation of trait polygenicity and more specifically for understanding the presence of a large number of false positives due to population structure. Chen et al. (2019), indicated the presence of secondary contacts between these main domains (Figure 3a), and current *P. abies* populations are mainly structured along a latitudinal gradient as are the climatic variables influencing growth traits (Figure 2, Figure 3b,c). In contrast, *bud‐burst* varies along both latitudinal and longitudinal gradients (Figure 3b). A lower confounding effect of population structure is thus expected for *bud‐burst* than for growth traits when investigating trait genetic architecture. In order to evaluate the impact of population structure on our ability to detect growth trait‐related SNPs, we reproduced the GWAS but without controlling for population structure (Figure 4). As expected for *bud‐burst*, *p*‐values were biased towards lower values, going down from 761 significant SNPs to 32, after correction for population structure and multiple testing. While significant and already rather massive, this effect was minor compared with the impact of population structure on SNPs associated with growth traits where the number of significant SNPs went from >50,000 without correction for population structure to ~180 with correction (Figure 4). Obviously, population structure impeded a proper detection of SNPs affecting growth traits in *P. abies*, because of likely over‐correction for population structure. It also means that our estimates of ~180 SNPs affecting growth traits is likely to be conservative and thus that the genetic architecture of *height* and *diameter* is highly polygenic. These results are in line with what was recently described for other quantitative traits in model species (e.g. Berg, Zhang, & Coop, 2017; Boyle et al., 2017; Daub et al., 2013), reconciliating genomic data with Fisher's infinitesimal model (Barton, Etheridge, & Véber, 2017; Turelli, 2017).

### 4.3 Southern genotypes outcompete local trees for growth traits

The strong association between genotype and phenotype variations showed that *height*, *diameter* and *bud‐burst* possess a strong genetic control. On the other hand, the association between genetic diversity and the environment of origin reflected a strong influence of population evolutionary history on genetic diversity. Finally, the fact that phenotypic traits followed environmental gradients revealed a strong pattern of local adaptation of Norway spruce populations to their original environment. Yet, despite this strong signature of local adaptation to the home environment, southern genotypes, for instance those from Romania, were taller and larger than northern ones when grown in southern Sweden although they resumed growth later in the spring than most northerly provenances. By linking phenotypic data to climatic variables, our study, as several before it (Dormling, 1979; Heide, 1974), highlights the importance of temperature in the control of *bud‐burst* and of, temperature, precipitation and photoperiod, on growth traits. Given climate change expectations in southern and central Sweden (+2 to 4°C annual mean temperature and higher annual precipitations, Swedish commission on climate & vulnerability, 2007), it will be crucial to consider the tree origins in future development of the breeding program in these regions.

Generally, transfer of trees within a range of four degrees of latitude is recommended to increase forest productivity (Persson & Persson, 1992; Rosvall, Andersson, & Ericsson, 1998), but transfer from farther provenance could, in contrast, lead to maladaptation (Savolainen et al., 2013 and references therein). A major limitation for assisted gene flow for boreal species come from the risk of frost damages due to late‐spring frost for northern genotypes (because of a too early *bud break*) or early fall frost for southern ones (because of a too long growth period, e.g. Montwé, Isaac‐Renton, Hamann, & Spiecker, 2018 but see MacLachlan, Wang, Hamann, Smets, & Aitken, 2017; MacLachlan, Yeaman, & Aitken, 2018 for lodgepole and interior spruce, respectively). In Norway spruce, the impact of frost damage on different provenances was investigated in a trial located in central Sweden by Hannerz & Westin (2005). They concluded that Belarus provenances were more productive than local ones but that the Belarus provenances also had an increased risk of autumn frost damage because of later hardening (Hannerz & Westin, 2005).

In the present case, frost damages were recorded within nine trials (135 trees) and no difference in frost resistance was observed between genetic clusters despite differences between trials (data not shown). However, the trees analysed in the present study are plus trees that were selected based on their superior phenotypes in southern Sweden. They are therefore not a random sample and, hence, we cannot draw inference on the effect of assisted gene flow from southern provenances in general. It is highly possible that ‘uncontrolled’ assisted gene flow could lead to an average decrease in growth performance and quality. Nonetheless, our data suggest that the samples that survive can have higher growth than local provenances. In the long term, episodic frost are expected to decrease in central and southern Sweden given global warming (Swedish commission on climate & vulnerability, 2007) even if frost damage risk could first increase during a transient period (Langvall, 2011). Given the superior productivity of more southerly provenances, assisted migration still appears as a strategy worth of further testing. In particular, biotic communities (insects, fungus and microbiome) and soil content represent two additional sources of maladaptation for large‐scale transfers that deserve further scrutiny (e.g. Aitken, Yeaman, Holliday, Wang, & Curtis‐McLane, 2008; Crémieux et al., 2008; Macel et al., 2007; Vitt, Havens, Kramer, Sollenberger, & Yates, 2010; Wang & Klinka, 1997). Indeed, in our different GWASs, we detected numerous genes involved in immune system responses as well as in metal‐ion transport or pH regulation. Investigating the relative impact of the afore‐mentioned risks would require additional studies to get a complete assessment of the effect of local soil and biotic communities on nonlocal genotypes.

Finally, thanks to large‐scale genomic data (>400 K SNPs), Chen et al. (2019) were able to characterize *P. abies* population structure at a finer scale than in previous studies. This indicated the existence of a new genetic cluster corresponding to hybrids between Fennoscandian and Alpine trees (CSE cluster). In the present study, we further showed that trees belonging to that cluster also had an intermediate phenotype evidencing that if some limitations to southern genotypes settlement exist they are clearly not strong enough to impede Alpine trees to reproduce with local ones. Hence, assisted gene flow would in the long‐term lead to a dilution of the local gene pool, a risk that should certainly be considered.

## 5 CONCLUSION

The sequencing of >1,500 Norway spruce trees coming from the Swedish breeding program allowed us to analyse the influence of tree origins on phenotypic traits and to investigate their genetic basis. From a practical point of view, our study lends support on strategies based on assisted gene flow to alleviate the impact of climate change in central and southern Sweden breeding program. First, trees with southern origin are taller and bigger than local ones (two valuable characteristics for the wood industry), and second, we showed that the control of these traits is highly polygenic, arguing for genomic selection approaches for trait improvement, especially considering the strong genetic correlation between both traits. From a more general perspective, our study revealed a strong pattern of local adaptation in Norway spruce, phenotypic traits following environmental gradients and tree origins explaining a large part of their variance. It also showed that breeding programs are valuable resources for large‐scale genomic studies. By tightly controlling environmental variance, they are ideal systems for investigations on the genetic basis of phenotypic traits. Re‐sequencing technologies being continuously developed and becoming more affordable, it will soon be possible to sequence a number of individuals large enough to apply statistical methods currently limited to humans and a handful of model species, allowing investigating, for instance, the strength and direction of selection acting on a trait of interest (Guo, Yang, & Visscher, 2018; Zeng et al., 2018 and references therein). Finally, our study showed that comparing traits that followed different geographic gradients could help to better comprehend and address the confounding effect of population structure on GWAS. Developing a statistical framework to control for population structure using this insight will, however, requires further investigation.

## CONFLICT OF INTEREST

The authors of this preprint declare that they have no financial conflict of interest with the content of this article.

## Data Availability

The data that support the findings of this study are openly available in DRYAD repository under https://doi.org/10.5061/dryad.mr47gq3 (Milesi et al., 2019).
